# Perspectives on advance research directives from individuals with mild cognitive impairment and family members: a qualitative interview study

**DOI:** 10.3389/fpsyt.2024.1419701

**Published:** 2024-09-20

**Authors:** Astrid Gieselmann, Jakov Gather, Marina Schmidt, Mishal Qubad, Jochen Vollmann, Matthé Scholten

**Affiliations:** ^1^ Ruhr University Bochum, Institute for Medical Ethics and History of Medicine, Bochum, Germany; ^2^ Charité – University Medicine Berlin, Department of Psychiatry and Psychotherapy, Berlin, Germany; ^3^ Ruhr University Bochum, Department of Psychiatry, Psychotherapy and Preventive Medicine, LWL University Hospital, Bochum, Germany; ^4^ Goethe University Frankurt/Main, Frankfurter Forum for Interdisciplinary Ageing Research (FFIA), Frankurt, Germany; ^5^ Goethe University Frankfurt/Main, Department of Psychiatry, Psychosomatic Medicine and Psychotherapy, Frankurt, Germany

**Keywords:** research ethics, dementia, vulnerability, mental capacity, substitute decision making, altruism, qualitative interviews

## Abstract

**Background:**

Advance research directives (ARDs) provide a promising way to involve individuals with mild cognitive impairment (MCI) in research decisions before they lose the capacity to consent. At the same time, the views of people with MCI on ARDs are underexplored. This study assesses the perceptions of people with MCI and family members on the benefits and challenges associated with ARDs.

**Aims:**

The aim of this study was to investigate the perspectives of individuals with MCI and family members of individuals with MCI on ARDs. We focus specifically on willingness to participate in nontherapeutic research, understanding of ARDs and the ethical considerations involved.

**Methods:**

Thirteen open-ended, face-to-face interviews were conducted using a semi-structured format. Seven interviews were conducted with individuals with MCI, and six with family members of individuals with MCI. The narratives were transcribed verbatim and qualitative content analysis was carried out.

**Results:**

Research participation and ARDs were viewed positively, largely based on altruistic motives and the desire to contribute to society. The participants recognized the potential advantages of ARDs in reducing the decision-making burden on family members and maintaining personal autonomy. They also highlighted challenges in comprehending ARDs and navigating the complexities surrounding potential conflicts between current preferences versus preferences described in an ARD.

**Conclusions:**

ARDs were predominantly seen as valuable instruments that enable individuals with MCI to participate in research. This study provides insights into the reasons why affected individuals are interested in drafting ARDs. These insights can guide the development of supportive interventions that are tailored to assist individuals with MCI and their families in navigating ARD processes.

## 1 Introduction

The prevalence of dementia is expected to rise dramatically as the global population ages. This development underscores the urgent need for advanced diagnostic and therapeutic options for cognitive impairments, notably, Alzheimer’s disease (1). Currently, there is a significant lack of effective treatments for Alzheimer´s disease, which makes research in this context and the development of therapeutic options even more important. Advance research directives (ARDs) have emerged as legal instruments that allow competent individuals to specify how research decisions should be made in the event that they lose the capacity to consent in the future. They have been proposed as a proactive approach for individuals who anticipate cognitive decline, such as those in the early stages of dementia, to prospectively consent to research participation (2).

Despite their potential benefits, ARDs are not yet commonly used in practice. Although several countries have established regulations governing their use, their adoption continues to be relatively low. A study from the US, for example, found that only 11% of the adult inpatients admitted to the NIH Clinical Center during the study period had completed an ARD, although nearly half of the participants were open to research that carried minimal risk without benefit (3). This discrepancy raises questions about the factors hindering the widespread adoption of ARDs, which may include a lack of awareness (4) or a preference to delegate such decisions to proxies (5).

ARDs are considered an extension of patient autonomy into future incapacity. Proponents of advance directives in the treatment context maintain that their ethical foundation lies in upholding the principle of respecting an individual’s precedent autonomy (6). In the research context, ARDs are viewed as a way to promote self-determination and express altruism (7).

Concerns have been raised, however, about the ability of ARDs to replace current informed consent. Furthermore, practical challenges related to ARDs, such as difficulties in predicting future research scenarios and ensuring that ARDs are specific enough to be applicable yet flexible enough to cover unforeseen research opportunities, have previously been discussed in the literature (8). ARDs have specifically been discussed as a means to preserve autonomy in the case of dementia and cognitive impairment (9). However, there is a concern that the use of ARDs in dementia research may lead researchers to overlook emotional expressions of participants who, due to their condition, may not be able to communicate effectively. This issue arises when an ARD contradicts current preferences (10).

Despite these challenges, researchers are willing to offer ARDs for various research protocols, indicating a potential shift towards their broader implementation (11). A recent qualitative study from the UK showed that stakeholders, including researchers, practitioners, and members of the public, generally support the concept of ARDs (12). At the same time, the effectiveness and acceptance of ARDs can vary significantly across different cultural and legal landscapes, which can impact perceptions and uptake (13). Empirical research to date has focused primarily on healthy research participants, neglecting the perspectives of those directly affected by mild cognitive impairment (MCI) and their families (14, 15). For this reason, it is crucial to gain a deeper understanding of how individuals with MCI perceive ARDs.

To our knowledge, only three studies have explored the perspectives on ARDs of individuals with MCI or family members. A randomized controlled trial by Stocking et al. (2007) involved 149 dyads and evaluated the utility of an ARD among persons with dementia and their proxies. The findings indicated no significant difference between the groups in terms of enrollment rates, decision-making ease, or proxy comfort (16). A study by Bravo et al. (2016) described an intervention that significantly increased the documentation of research preferences among elderly participants. In their randomized controlled trial conducted in Canada, 80% of participants in the intervention group completed an ARD, a much higher rate compared to those who did not receive the intervention (17).

These results suggest that while ARDs may be feasible, their practical impact on decision-making in dementia research remains uncertain. This underscores the need for further investigation into how individuals with MCI and their families perceive ARDs. In addition to that, it seems important to evaluate the perspectives of persons with MCI also using other methodologies, such as qualitative methodology, and in different geographical areas.

To date, only one study was set in Germany and used similar methodology (18). Jongsma et al. (2020) have addressed the motivations and concerns of individuals with MCI regarding ARDs. This study involved semi-structured interviews with 24 participants who expressed a predominantly positive view towards ARDs. Participants highlighted the importance of drafting ARDs at an early stage. Building upon these findings, our study explores how individuals with MCI and their family members evaluate the use of ARDs, especially in terms of their willingness to participate in nontherapeutic research. While Jongsma et al. (2020) found a generally positive attitude toward ARDs among participants, our study aimed to verify these views, further investigate ethical considerations and extend the focus to include the perspectives of family members.

## 2 Method

This study used qualitative, semi-structured interviews to investigate the motivations and perceptions of individuals with MCI and their family members towards ARDs. The objective was to comprehend their willingness to participate in research, their understanding of ARDs, and views on the ethical and practical issues surrounding ARD implementation. Thirteen participants, seven persons diagnosed with MCI and six family members of persons with MCI, were interviewed. Some of the interviews were conducted jointly and the rest independently by A.G. and M.Schmi.

The majority of the participants with MCI and family members were identified and recruited through collaboration with a resident in psychiatry (M.Q.) at the University Hospital of Psychiatry in Frankfurt, Germany. This approach ensured access to persons who met our inclusion criteria which was having a confirmed MCI diagnosis. In addition, we recruited two participants, one person with MCI and a family member through a facilitated self-support group for individuals with dementia and their caregivers. The aim was to incorporate views from individuals who are actively seeking community support. This recruitment channel facilitated the inclusion of additional perspectives in our study.

Demographic and professional background details were collected from each participant, including age, gender, nationality and previous profession, in order to assemble a diverse sample. We included three female and four male individuals with an MCI diagnosis, ranging in age from 62 to 85 years. Four of the participants were related. One dyad consisted of a married couple, while the other comprised a mother and her daughter. The remaining participants were not related to each other. The family members consisted of four females and two males, aged between 55 and 78 years. Participants were informed about the study’s scope and process before participating. Written informed consent was obtained from all participants. None of them were familiar with the concept of ARDs before their involvement in the study.

Data was collected through semi-structured interviews conducted in Frankfurt between January and August 2019. The interviews followed a topic guide that was developed based on a review of relevant literature and ongoing ethical and political discussions surrounding ARDs. The guide sparked discussions on critical topics, including the aims of biomedical research, distinctions between therapeutic and nontherapeutic research, the concept of “minimal risk” in nontherapeutic research, comprehension of ARDs, and the potential benefits and risks associated with ARDs.

To familiarize participants with the concept of ARDs, they were briefed on Germany’s legal framework governing ARDs at the beginning of the interview. In Germany, the Fourth Amendment to the German Medicinal Products Act (*Arzneimittelgesetz*), adopted on November 11, 2016, allows nontherapeutic biomedical research with individuals who are unable to give consent under conditions specified in EU Regulation No. 536/2014. In addition to these conditions, the Fourth Amendment to the German Medicinal Products Act introduced ARDs as an additional safeguard, requiring individuals to explicitly declare their willingness to participate in nontherapeutic research (19, 20).

The interview guide allowed for spontaneous follow-up questions to ensure rich and in-depth data collection. Supported decision-making tools in the form of cards featuring key terms related to the study’s themes were used to enhance the comprehension of ARDs and facilitate discussion. Interviews were conducted in settings convenient for the participants, either at the hospital or in their homes. All interview sessions were audio-recorded and transcribed verbatim. The interviews varied in duration, with the shortest being 21 minutes and the longest 47 minutes, while the average duration was 32 minutes.

The data was subjected to a systematic analysis employing thematic analysis methodology, as outlined by Braun and Clarke (21). Initially, deductive coding was employed using an initial coding framework based on the existing literature. This initial phase facilitated the structuring of the analysis around specific, anticipated themes, including the perception of ARDs, the ethical considerations in research, and the understanding of non-therapeutic research involvement. Subsequently, inductive coding was employed to identify additional themes. The coding process was iterative, with codes being continuously reorganized as new data were analyzed. All interview transcripts were coded by A.G. and the resulting code structure was discussed in team meetings with J.G. and M.Scho.

The research protocol, including the recruitment strategy, informed consent process and data handling procedures, was approved by the Research Ethics Committee of the Ruhr-University Bochum (No. 17-6145-BR).

## 3 Results

The socio-demographic characteristics of the participants are presented in **Table 1**. A pivotal finding of this study was the predominantly positive attitudes of participants towards research participation as such and the potential of ARDs in their own lives. It is noteworthy that the participants were unfamiliar with the concept of ARDs prior to their involvement in this study, and many participants faced challenges in understanding the complexities of ARDs. The following sections will delineate the nine central themes identified through detailed analysis of the narratives of people with MCI and their family members. Each theme will be illustrated with exemplifying quotes.

**Table 1:** Socio-demographic characteristics of people with MCI and family members.

Characteristics	People with MCI	Family members
**Number of participants**	7	6
Gender		
Male	4	2
Female	3	4
Age		
50-59	0	2
60-69	2	2
70-79	2	2
80-89	3	0

### 3.1 Positive outlook on research participation

Our findings revealed that most participants held positive attitudes towards research and the idea of contributing to it. Participants articulated several motivations for engaging in research, with the desire to contribute to the broader societal good being the most compelling driver. This altruistic inclination was characterized by their desire to help researchers gain “*new insights*” (Person with MCI 5), thus, supporting the advancement of medical science. Furthermore, some people with MCI expressed optimism that their involvement in research could eventually lead to the discovery of a cure for their condition.

### 3.2 Altruistic motivations for research participation

A significant emphasis in our study was placed on understanding participants’ attitudes towards nontherapeutic research, which aims to benefit society as a whole rather than the individual participant. When the distinction between therapeutic and nontherapeutic research was clarified, especially in instances where participants did not initially mention the potential for personal benefit, the altruistic motive for participating in research emerged prominently in the discussions. Most participants expressed their willingness to engage in research if it meant the possibility of aiding others. One participant stated, “*If I can help others by doing it, I’m happy to do it*” (Person with MCI 4). This highlights a prevalent willingness to contribute to the welfare of others beyond personal gain.

This theme extended to a more familial and generational perspective, with participants acknowledging the significance of research in its potential to safeguard the future health of their family members and descendants. One participant, for example, reflected on the hereditary aspects of MCI and expressed a proactive stance towards contributing to research with the hope of contributing to advancements that could benefit their children and grandchildren. The participant stated, “*For me, it is logical that one tries to get the best out of research for oneself, and for others, of course, I have children, I have grandchildren, I want them to be well, and especially if I think about dementia, it might be hereditary, at least from what I’ve heard, so I’m glad, if research at some point would be able to treat the illness*” (Person with MCI 7).

### 3.3 Acceptable interventions in nontherapeutic research

In our investigation we inquired about the willingness of participants to engage in nontherapeutic research and their comfort levels with various research interventions, particularly those classified as carrying minimal risk and burden. These interventions typically involve questionnaires, interviews, physical measurements (e.g. of weight and height), blood draws and noninvasive diagnostic measures, such as an electrocardiogram or electroencephalogram.

Most persons with MCI assessed questionnaires, interviews and physical measurements as relatively nonintrusive and noninvasive methods carrying minimal risk and burden. These interventions were generally perceived as neither problematic nor burdensome. A family member stated, “*counting, measuring and weighing: It doesn’t hurt me. If you need it, please, you are welcome to have it*” (Family member 2).

This perspective was further supported by a family member who argued that individuals with MCI who can participate in an interview can discontinue research participation if they choose, highlighting the nonintrusive nature of such methods.

Opinions were divided regarding procedures involving blood draws and imaging techniques, with some participants not perceiving them as particularly burdensome, while others expressed reservations. One person with MCI explicitly stated their opposition to blood draws, saying, “*no, for research I wouldn’t let someone draw blood from me*” (Person with MCI 4). This difference in opinions highlights the participants’ varied understanding of what are considered acceptable burdens and risks in the context of nontherapeutic research.

### 3.4 Grasping the concept of an ARD

Participants first became familiar with the concept of ARDs during the interviews. Despite their familiarity with advance directives for health care – a concept many had not only heard of but had also had concrete experience with – participants encountered significant challenges in understanding the nuances of ARDs, such as the idea of planning for hypothetical research participation. The concept of ARDs was entirely new to all interviewees prior to their participation in the study. One person with MCI stated: “*actually, I never thought about this before*” (Person with MCI 1).

The difficulty in understanding ARDs was not equally distributed across participants. Family members of individuals with MCI generally exhibited a clearer grasp of the concept, while people diagnosed with MCI themselves faced more challenges, such as understanding complex ideas like “informed consent”. This highlights the cognitive demands involved in conceptualizing ARDs, particularly for those directly affected by cognitive impairments.

### 3.5 Advantages of ARDs

The discussion on the benefits and drawbacks of implementing ARDs surfaced several key points. Persons with MCI and their family members identified potential advantages, with a principal benefit of ARDs being the reduction of decision-making burdens on family members. One individual with MCI stated, “*I don’t want that they, yes, for the family members it is relatively hard to decide, yes, and if I decide beforehand, when I decide beforehand myself that I don’t want this or that, then it will be easier for them*” (Person with MCI 2).

The aspect of maintaining control over personal decisions, specifically in the absence of decision-making capacity, was another critical advantage underscored by participants. A representative quote from the discussions illuminates this: “*The advantage is that I decide about my, about my life, and I think that’s okay*” (Person with MCI 2). This perspective underlines the importance of ARDs in preserving self-determination.

Moreover, participants identified ARDs as a vital tool to enable research that otherwise might not be possible due to the difficulty in obtaining current informed consent, particularly in later stages of conditions such as Alzheimer’s disease. A family member observed, “*because I do see that in case of a patient with Alzheimer disease in a late stage, research could not be done otherwise, if he wouldn’t have given his informed consent, so this is an aspect that I do think is important, yes*” (Family member 5).

### 3.6 Potential disadvantages of ARDs and concerns about research participation

Although participants mostly emphasized the advantages of ARDs, some expressed concerns that shed light on potential disadvantages. The refusal to participate in research by means of an ARD was often linked to a general fear of participating in research. A woman with MCI, for example, expressed this by stating, “*I don’t want to say anything in advance, that later I can’t stand by*” (Person with MCI 4).

Participants also expressed concerns about the possibility of changing their minds in the future, highlighting the difficulties around deciding well in advance of a study’s start. A family member stated: *“Yes, whether that really still is the will in that situation is very questionable. Because my will would probably change then too. You often say in theory that if this or that happens, then I definitely want to die. And many people then say: Oh no, I actually don’t want to. I always say, it’s theory and practice. You have to experience it first before you can really give your judgment on it. It’s very, very difficult.”* (Family member 1). Additionally, worries about possible future regret were prominent.

The discourse highlighted the challenges that ARDs may pose for family members, particularly when their views diverge from the directives. One family member expressed concern about the potential conflict this could cause: “*If I would judge the situation differently in that moment, then it wouldn’t be helpful, and then, then maybe it would put me under pressure, he wanted it differently, but I think it’s not good for him anymore, that could put me in a dilemma [ … ] that could be a disadvantage then*” (Family member 1).

Another layer of complexity is introduced when considering the impact of an ARD decision on caregivers. A family member put herself in the situation of being diagnosed with MCI herself and commented, “*If I would agree to that, then I would indirectly also compel the person who takes care of me to carry the burden of participation*” (Family member 5). This comment underscores the ripple effect of an ARD decision, extending its impact beyond the individual to those tasked with their care.

### 3.7 Trust in family members regarding decision-making

A significant and unprompted theme emerged during our discussions regarding the trust affected individuals place in their family members to make decisions on their behalf. Many participants expressed a preference for familial decision-making over documenting their own choices in an ARD. One participant stated that he trusted his wife’s judgment more than his own due to potential changes in personality over time: “*as I might have written it ten years ago, then I would have been a completely different person, and now, now I am an old person, I might have a lot of experience, and my wife knows my experience, then I, then I need the experience or the decision of my wife*” (Person with MCI 6).

Similarly, another participant placed her confidence in her daughter’s professional judgment, attributing to her the responsibility to make the best decisions in the event of any health-related issues: “*My daughter is a doctor, and if something happens to me, then she will find the right way. I leave it to the children to decide what should be done*” (Person with MCI 4).

### 3.8 Importance of information and communication during ARD development

During the interviews, participants discussed crucial factors in drafting an ARD. The need for professional guidance was emphasized, with many participants expressing a desire for, or even considering it essential to have, a consultation with a physician during the ARD drafting process. This preference underscores the importance of expert advice when dealing with the complexities of ARDs and making informed decisions.

The specificity of ARD content was also a topic of discussion. Participants varied in their views, but there was a consensus on the need for precision in the directives, while also considering the limitations of laypeople in understanding the specifics of research protocols. A family member expressed this by highlighting the need for clarity and guidance: “*That should be relatively precise, but for me, who is not a researcher, I wouldn’t know how to formulate that in detail [ … ] the researcher would have to know that [ … ] then I would be presented the catalogue and I could say I want this, but I don’t want that*” (Family member 2).

### 3.9 Navigating conflicts between ARDs and current preferences

The interviews ended with a discussion on scenarios where conflicts arise between the preferences described in an ARD and the current preferences of a person with MCI. This conversation was sparked by a hypothetical case in which a person with MCI who had previously consented to research participation by means of an ARD subsequently exhibited reluctance when approached for a study procedure.

Participants demonstrated a nuanced understanding of the conflict presented, despite initial difficulties with the concept of ARDs. The consensus leaned towards respecting the affected individual’s present dissent, emphasizing the ethical principle of autonomy. This stance was expressed by an individual with MCI: “*I think, pulling the arm away and then taking blood, I don’t like that*” (Person with MCI 2). Another participant with MCI argued: “*First, one would have to speak with her, with the person, you can’t do anything with force there*” (Person with MCI 5).

Participants emphasized the importance of respecting the current preferences of individuals who lack decision-making capacity by highlighting their vulnerability and argued for exclusion from research if individuals are not able to articulate preferences anymore. One person with MCI stated, “*He basically can’t speak anymore, and*, *therefore, can’t justify himself, I would say, and that’s why one is not allowed to do that*” (Person with MCI 1). Another family member suggested that the research could proceed with other participants to minimize the need to enforce participation against an individual’s current preferences.

The conversation also addressed the fact that people in advanced stages of dementia retain their preferences, which may evolve over time. One family member argued, “*a person with severe dementia has a will, too, is not without a will but maybe with a different will than the one he had declared two, three, four years ago*” (Family member 5).

At the same time, a recurring theme was the possibility of reengaging with the individual at a later time, acknowledging the fluctuating cognitive abilities associated with dementia. This approach demonstrates the need for a balance between honoring immediate expressions of preferences and recognizing the potential for change over time.

## 4 Discussion

Research involving individuals who are deemed incapable of giving consent remains a highly debated and challenging issue. ARDs have been suggested as a way to resolve this ethical dilemma. The participants in this study frequently expressed positive attitudes towards research participation, which confirms previous research findings (18, 22). Many of the participants’ responses suggest that altruism and a desire to contribute to societal good are motivating factors. Based on the views expressed by participants, ARDs may offer a way to maintain personal autonomy and could potentially reduce decision-making burdens on family members. Some of the participants also perceived these directives as potentially facilitating important research.

Within the ethical and political discourse surrounding ARDs, policy-makers, dementia researchers and ethicists have made assumptions about the perspectives of people with MCI towards ARDs. Our findings support some of these presuppositions. Previous research (7, 18, 23) has suggested that individuals may be motivated to participate in nontherapeutic research out of altruism or the hope that such research could benefit future generations. Consistent with these findings, many interviewees in our study expressed a desire to contribute to the well-being of others through research participation. Furthermore, some participants hoped that their involvement could potentially benefit their descendants, who may be at an increased risk of developing dementia. A distinctive insight from our study is the prioritization of altruistic motivations over the principle of self-determination in the drafting of ARDs by affected individuals. While theoretical discussions on ARDs (24) value these instruments primarily because they enhance patient autonomy and self-determination, these concepts were found to be of secondary importance to the participants in our study.

However, it is important to question whether all forms of participation driven by a desire to contribute to the well-being of others truly qualify as altruistic (25). Participants may perceive personal indirect benefits, such as a sense of purpose or emotional satisfaction from believing that their actions could benefit future generations. This introduces a potential overlap between altruistic motivations and self-interest, suggesting that what might initially appear as altruism could also partly serve the participants’ psychological or social needs.

Another finding of our study is the apparent reluctance among some participants with MCI to commit to decisions via ARDs which they might not be able to uphold in the future. This observation appears to diverge from literature on advance care planning (ACP) for treatment decisions, which documents a generally favorable disposition towards ACP among service users and professionals alike (26). This discrepancy may be attributed to differences between advance care planning for research and advance care planning for health care. ARDs in the context of research involve decisions about participation in future studies that might be unfamiliar at the time of decision-making, in contrast to ACP, which often concerns more immediate medical treatments.

The discourse on the benefits and drawbacks of ARDs revealed varied perspectives among participants on the delegation of decisions about research participation. A significant theme that emerged was the consideration of an alternative to ARDs, where a family member or legal authorized representative makes decisions regarding research involvement. Some argued that ARDs can alleviate the decision-making burden on family members, positing that ARDs serve as a solution that could simplify difficult decisions. Others believed that family members may be better equipped to make decisions about research participation than the individuals themselves at the time of drafting the directive. The theme of trust in proxy decision-makers aligns with prior research that found a general trust in the decision-making abilities of family members on behalf of affected individuals (27). Research also indicates, however, that proxies may not always accurately predict the preferences of the individuals they represent (28, 29). This raises concerns about the reliability of family members or legally authorized representatives in making decisions that align with the affected individual’s wishes. While the preference to delegate decision-making to family members exists, an ARD may, therefore, offer a more precise reflection of the individual’s preferences. This precision underscores the potential value of ARDs in ensuring that research participation decisions are more closely aligned with the affected individual’s autonomous choices.

The definition of “minimal risk” and “minimal burden” is a controversial topic in research ethics (30). This issue is also crucial in Germany, where nontherapeutic research in noncompetent populations is only allowed under these conditions and prior consent in an ARD (19, 20). German legislation, however, does not provide a clear definition of these terms, which creates a significant gap in guidance for researchers, counselors and participants. Participants in this study considered activities such as completing questionnaires, participating in interviews and basic physical measurements as carrying minimal risk and burden. More invasive or intrusive procedures, such as blood draws and the use of imaging technologies, elicited varied responses, highlighting the subjective nature of perceived risk and burden among individuals. Furthermore, the results indicate a clear preference among some potential research participants towards avoiding procedures deemed to exceed minimal risk and burden. Our findings, thus, support previous recommendations that a well-designed ARD should provide a detailed account of various research activities that refine a person’s preferences and risk tolerances (31).

The necessity of mandatory counseling prior to the drafting of an ARD has emerged as a significant concern in the discourse surrounding ARDs (20). Our interviews indicate a strong preference among participants for information disclosure provided by physicians. Furthermore, our findings reveal that individuals with MCI find the concept of ARDs and the deliberation about their research participation preferences to be particularly challenging. Consequently, these insights indicate that information disclosure is essential, for both practical and ethical reasons, to ensure that individuals are fully informed and able to make decisions that accurately reflect their wishes and interests. Our findings also suggest that potential research subjects would accept a practical disclosure standard which has been proposed for ARDs (20, 32). In order to inform potential research participants about studies, researchers could describe types of research studies that pose minimal risk and burden, rather than providing information about specific studies (33). This would include information about potential research studies that have not been designed yet.

A longstanding ethical dilemma discussed in existing literature regarding the use of advance directives, particularly in case of dementia, involves the tension between the preference described in an advance directive and an individual’s current preferences (10, 34). When the German legislation concerning ARDs was drafted, lawmakers explicitly stated that the current preferences of the individual should always take precedence. A pressing and unresolved practical question, however, is how to interpret and apply this principle in everyday research contexts. Specifically, it remains unclear what types of expressions from a research participant who lacks decision-making capacity should be interpreted as refusal to participate in research or as a withdrawal of consent given previously (35). Our study reveals that affected individuals and their family members have concerns about the potential for research participation to proceed contrary to a person’s current wishes. These concerns align with the attitudes of researchers. Researchers in a previous study agreed strongly that current dissent of a research participant should take precedence over their previous consent as stated in an ARD (11). Our results also underscore the need for a minimal threshold for expressing dissent. This means that even nonverbal cues indicating an individual’s reluctance or withdrawal should be respected.

Another important aspect to consider is the potential for regret, which is associated with all forms of advance directives. There remains the possibility that individuals may feel differently about decisions they made in an ARD at a later point. It is therefore important to establish mechanisms that allow for reviews and revision of ARDs over time.

The findings of our study are subject to certain limitations. Given the complexity of the concept of ARDs and the inherent challenges it posed to affected individuals and their family members, we used supported decision-making tools (36), including cards featuring key terms, to facilitate understanding. While these aids improved participants’ comprehension of the questions, they may have introduced a bias and influenced their responses.

In addition to that, the small sample size of thirteen participants limits the generalizability of our findings.

The selection of participants may have introduced bias as well. The sample was predominantly recruited from a single urban hospital in Frankfurt, Germany. The urban setting of the study may influence the participants´ experiences, as urban populations often have better healthcare access and more progressive views than people in rural areas. In addition to that, individuals who are willing to discuss their preferences for decision-making around research participation might have more defined views on the subject, which could steer the findings towards those with stronger opinions or more positive attitudes toward ARDs. Moreover, the inclusion of dyads (a married couple and a mother and her daughter) likely influenced the discussions about ARDs, as these participants may have shared mutual expectations about each other´s preferences and values.

The temporal gap between data collection and the current date limits the relevance of our findings for the parliamentary discussion around ARDs in Germany, as the Fourth Amendment to the German Medicinal Products Act, which includes legal provisions for ARDs, was passed in parliament in 2016. At the same time, implementation of ARDs has since progressed slowly, and it is unlikely that the fundamental ethical issues surrounding ARDs and the attitudes of stakeholders towards these issues have changed significantly in the meantime. The findings thus remain relevant. Moreover, many jurisdictions worldwide do not have legal provisions for ARDs. The findings from this study can hence inform policy-making in these jurisdictions.

## 5 Conclusion

ARDs represent a potentially valuable mechanism for ethically facilitating the participation of individuals with MCI in research. At the same time, the deployment of ARDs raises ethical challenges. Our investigation shows that both individuals with MCI and their family members recognize the significance of dementia research and are willing to participate in research by means of ARDs. Although the concept of ARDs was new to participants, they recognized their potential to maintain personal autonomy, reduce decision-making burdens on family members and facilitate crucial research in dementia.

However, our study also highlights the challenges and ethical issues surrounding ARDs, such as the difficulty comprehending their concept, the possibility of changing preferences and the importance of clear communication. The necessity of professional guidance was emphasized by individuals with MCI and their family members alike. Our findings, therefore, support previous recommendations to develop training and educational resources for researchers, ethics committees and organizations to enhance their readiness to involve people with MCI in research (37).

Counseling could play an essential role in this context. Experience from advance directives for healthcare underline the importance of communication and support in the decision-making process. This can be applied to ARDs as well. In addition to physicians, other healthcare professionals and trained counselors could be responsible for counseling.

In order to minimize the challenges and barriers of ARD utilization, our study highlights the need for targeted interventions aimed at facilitating clear communication to ensure that individuals fully understand ARDs. Standardized templates, which have been suggested previously (18), could help address concerns about the complexity of drafting ARDs. By addressing these needs, it is possible to enhance the ethical quality of dementia research and ensure that the voices of those most affected are heard and respected.

## Data Availability

Due to the specifics of the informed consent obtained from participants, the interview transcripts are not available for sharing in their entirety. Participants consented only to the use of anonymized quotes from their interviews in the publication. Selected, anonymized excerpts can be provided upon reasonable request where they do not violate the terms of consent. Requests to access the datasets should be directed to astrid.gieselmann@charite.de.
