# Supplementary material for: Perspectives on advance research directives from individuals with mild cognitive impairment and family members: a qualitative interview study
Source: Front Psychiatry. 2024 Sep 20;15:1419701. doi: 10.3389/fpsyt.2024.1419701 (PMC11450480; doi:10.3389/fpsyt.2024.1419701)
Supplement: Supplementary file 1 [file DataSheet1.docx]

**Supplementary materials**

For the article: “Perspectives on Advance Research Directives from Individuals with Mild Cognitive Impairment and Family Members: A Qualitative Interview Study”

Please note that the original materials were in German. These have been translated into English for reasons of transparency.

**1. Interview guide**

| **Guide** | **Checklist** | **Concrete questions – when key questions remain unanswered** | | **Maintenance and control questions** |
| --- | --- | --- | --- | --- |
| **1. Introduction** | | | | |
| Saying thank you for taking part in the study | | | | |
| Presentation | | | | |
| Explanation of the procedure: confidential, anonymous, topic, time approx. 60 min, informing about the recording | | | | |
| Organizational issues | | | | |
| **2.** **Questions about previous experience and knowledge**  *In what follows, we will first clarify a few terms that are important for understanding this study. There is no right or wrong. It is about how you understand the terms and I will then explain again how we researchers understand the terms. This is important so that we both know what we are actually talking about.* | | | | |
| **What do you understand by an *advance directive*?** | *Time, preferences*  *(Declaration of intent for a time when a person is no longer has capacity to consent.)*  *Medical measures* | Do you have an advance directive? (Yes/No)  Why (not)? | | Can you elaborate? |
| **Explanation if applicable:** An advance directive is a document that is usually used to document preferences regarding treatment at the end of life. At the end of life, people often find themselves in situations in which they no longer understand important information or are no longer able to express themselves. In an advance directive, people can write down how they want to be treated in such situations. | | | | |
| **Are you familiar with the fact that that there is research that benefits others rather than the research participants themselves?** | *Benefit -> Group*  *(If research benefits a population group to which the patient belongs [e.g. people with dementia])*  *therapeutic research vs. non-therapeutic research*  *group benefit vs. individual benefit* | *Have you heard about it?*  *How/where did you hear about it?* | | What do you think is meant by this? |
| In research, a fundamental distinction must be made between research that benefits the research participants themselves and research that does not benefit the research participants themselves.  Research that does not benefit the research participants themselves but can benefit other people with the same medical condition (e.g. dementia) in the future is referred to as non-therapeutic research. | | | | |
| **What do you understand by an advance research directive?** |  | *Have you heard about it?*  *How/where did you hear about it?* | | What do you think is meant by an advance research directive? |
| An advance research directive is an advance directive for potential future research participation. In the later stages of dementia, the person may no longer be able to give consent to research participation.  The idea behind the advance research directive is that the person can specify at the onset of dementia in an advance directive whether they wish to participate in research at a later stage. | | | | |
| In Germany, people with dementia are allowed to write an advance research directive. In the advance research directive, they can declare whether or not they wish to participate in research at a later point of time.  They can then also take part in research that is of no benefit to them. However, they can only take part in research that involves minimal burden and risk.  Research with minimal burdens and risks involves research measures such as surveys, interviews, measurements and weighing.  Are you aware of this? Do you have any questions about this? | | | | |
| **3. Questions on the ethical and practical aspects of advance research directives and non-therapeutic research with individuals with dementia who are unable to give consent** | | | | |
| **Would you participate in research that has a benefit for yourself?** | *Yes*    *No*  *Reasons* | Why?  Under what circumstances?  What would be your motivation?  What would you hope to achieve? | | Can you explain your attitude in more detail?  Can you give reasons?  Please take your time. |
| **Would you participate in research that has NO direct benefit for you?** | *Yes*  *No*  *Reasons* | Why?  Under what circumstances?  What would be your motivation?  Would you also think of your own relatives?  What do you hope to achieve? | | Can you explain your attitude in more detail?  Can you give reasons?  Please take your time. |
| **Would it make a difference if this research could help others?** | *Yes*  *No*  *Reasons* | Why?  What would be your motivation? | | Can you explain your attitude in more detail?  Can you give reasons?  Please take your time. |
| **Imagine you were taking part in research that only benefited others.**  **What measures would you then accept?** | *Give examples:*  *- interview*  *- questionnaire*  *- weighing*  *- measuring*  *- blood sampling*  *- imaging procedures*  *- medication* | Why?  What would be your motivation?  Where would you draw the line? | | Can you explain your attitude in more detail?  Can you give reasons?  Please take your time. |
| **Which advantages and disadvantages do you see in relation to advance research directives?** | *Participation also possible when a person is unable to consent*  *Pressure to participate in research* | *In your opinion, could problems arise when one where to implement advance research directives?*  *If so, what are your concerns?* | | Please take your time to think about it.  Could you try to explain to me what would be good or bad about an advance research directive? |
| **On the drafting of the advance directive:**  **In your opinion, how should the advance research directive be drafted?** | *Concrete*  *Risk* | *How specific and detailed should it be?*  *Should the specific study be documented in the directive or only certain types of studies (e.g. interview studies)?* | | Please take your time to think about this. |
| **On counselling:**  **Would you like advice on drafting an advance research directive?** | *Yes*  *No*  *Reasons* | *What should the doctor explain?*  *What would be helpful for you during the explanation?*  *Should the explanation be supported by materials, e.g. images?*  *What concerns would you have?* | | Can you explain your attitude in more detail?  Please take your time |
| **On the implementation of the advance directive:**  **The issue now is not the drafting, but the implementation of the advance research directive.**  **Should the advance research directive be taken into account in all cases?** | *Yes*  *No*  *Reasons* | *What would be reasons not to comply with the advance research directive?* | | Please take your time to think about this. |
| **Case study for implementation:**  **Imagine the following example. A person with mild dementia has drawn up an advance research directive. In the advance research directive, he has agreed to take part in certain studies. He has agreed to take part in questionnaire studies. Five years later, his dementia has progressed. During a hospital stay, a doctor comes into his room in the morning and wants to fill out a questionnaire with him. At this point in time, however, the patient is not in the mood. The patient has difficulty communicating this to the doctor verbally. However, he turns away from the doctor. The doctor therefore assumes that the patient is not in the mood.**  **Should the person be included in the study?**  **Or should he be excluded?** | | *Why?*  *What other behaviors would be a reason not to conduct the study? E.g. head shaking, bad mood* | Can you explain your attitude in more detail?  Please take your time. | |
| **In your opinion, what may the doctor do to try to get the person to take part in the study after all?** |  | *May he "persuade" the person?*  *May he come back at a later point in time?* | | Try to put yourself in the situation.  Please take your time to do so. |
| **4. Final questions** | | | | |
| **Do you still have questions?** |  |  | |  |
| **Did I miss mentioning something that is important to you?** |  |  | |  |
| **How did you experience the interview?** |  |  | |  |
| **5. Socio-demographic information & other** | | | | |
| **(*Sex: noting independently*)** | *- male*  *- female*  *- diverse* |  | |  |
| **What’s your nationality?** | *- German*  *- Other* |  | |  |
| **What’s your native language?** | *- German*  *- Other* |  | |  |
| **What’s your marital status?** | *- Married*  *- Unmarried*  *- Other* |  | |  |
| **How old are you?** | *- Age* |  | |  |
| ***Have you already taken part in research projects on dementia?*** | *If so, how often?* |  | |  |
| **Profession / former profession?** |  |  | |  |

**2. Key word lists and memo cards**

Please see the following pages.

**Keyword list minimal risk**

- Interview
- Questionnaire
- Weighing
- Measuring
- Blood sample
- Radiological imaging

**Keyword list advance directive**

- Document
- Medical treatment
- End of life
- Can no longer decide for yourself
- Write down your wishes in advance
- Application: when you are no longer able to decide for yourself

**Keyword list advance research directive**

- Document
- Participation in research
- Write down your wishes in advance
- Application: when you are no longer able to decide for yourself

**Keyword list case study**

- Person with early-stage dementia
- Advance research directive: participation in research
- Later: severe dementia
- Application of the advance research directive
- Doctor wants to fill out a questionnaire with the person
- Person turns away
- Doctor believes patient does not want to participate
